# Comparative Analysis of Mitochondrial Genomes and Phylogeny of Barbastelle Bats Across China

**DOI:** 10.1002/ece3.72949

**Published:** 2026-01-12

**Authors:** Sen Liu, Xinyu Yue, Hang Li, Yangguang Sun, Xufan Wang, Dongge Guo, Ying Wang

**Affiliations:** ^1^ College of Life Sciences Henan Normal University Xinxiang Henan China; ^2^ The Observation and Research Field Station of Taihang Mountain Forest Ecosystems of Henan Province Xinxiang Henan China; ^3^ North Henan Medical University Xinxiang Henan China

**Keywords:** *Barbastella beijingensis*, *Barbastella darjelingensis*, barbastelle, mitochondrial genome, phylogenetic analysis

## Abstract

Barbastelle bats are notable for their nearly square outer ears that converge at the forehead. Knowledge of their mitochondrial genome is limited. This study sequenced the complete mitochondrial genomes of *Barbastella beijingensis* and *B. darjelingensis*, the latter being reported for the first time. Subsequently, we performed a comprehensive comparative analysis with the published mitochondrial genome of *B. capsica*. The mitochondrial genomes measured 16,667 bp and 16,434 bp, respectively, and included 13 protein‐coding genes (PCGs), 22 tRNA genes, 2 rRNA genes, and one D‐loop. Their gene order matched other bat species in the Vespertilionidae and Phyllostomidae families. All three species showed low *K*_*a*_/*K*_*s*_ ratios in the PCGs, indicating strong purifying selection, especially in cytochrome oxidase respiratory chain complex (*COX1*, *COX2*, and *COX3*), while *ATP8* faced relatively relaxed selective pressure. In all three species, tRNAs showed typical cloverleaf structures, except for *trnS1*, which lacked a D arm. The D‐loop region is divided into ETAS, CD, and CSB domains. Both ETAS domains have ETAS1 and ETAS2 motifs, and the CD domain contains conserved motifs box B–F. While CSB1–CSB3 were conserved in *B. capsica*, only CSB1 was found in *B. beijingensis* and *B. darjelingensis.* Phylogenetic analysis of the 13 PCGs strongly confirmed the three barbastelle species as distinct within *Barbastella*, closely related to genera *Plecotus* and *Corynorhinus* in the Plecotini tribe. This study enhances the molecular data available for the genus *Barbastella* and offers essential mitochondrial genomic evidence to resolve persistent taxonomic disputes within the Vespertilionidae family.

## Graphical abstract

Barbastelle bats are characterized by their distinct approximately square‐shaped outer ears, which converge at the forehead. In China, three species have been documented, and we present two mitochondrial genomes for *Barbastella beijingensis* and B. darjelingensis. Every mitochondrial genome is composed of 13 protein‐coding genes (PCGs), 22 transfer RNA genes, two ribosomal RNA genes, and one D‐loop. Phylogenetic analysis based on the 13 PCGs strongly supported *B. beijingensis* and *B. darjelingensis* as distinct species within the genus *Barbastella*, which exhibits relatively close phylogenetic relationships with the genera *Plecotus* and *Corynorhinus* within the family Vespertilionidae.

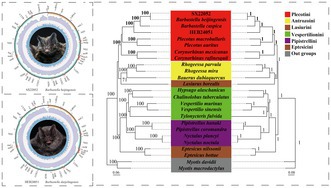

## 1 Introdution

Within the Vespertilionidae family of the Chiroptera order, the phylogenetic relationships among numerous genera remain highly contentious, particularly for taxa characterized by restricted distribution ranges and limited sampling data, such as the Barbastelle bats. These bats are distinguished by their almost square outer ears, which are connected at the forehead by a skin flap and exhibit prominent transverse skin folds on the midsection of the pinna. The *Barbastella* genus comprises six species: *Barbastella barbastellus* , *B. leucomelas* , *B. capsica*, *B. darjelingensis*, *B. beijingensis* , and *B. pacifica* (Zhang et al. 2007; Benda and Mlikovsky 2008; Kruskop et al. 2019; Russo et al. 2023; Wang and Abduriyim 2025). Traditionally, *Barbastella* with *Corynorhinus*, *Euderma*, *Idionycteris*, and *Plecotus* has been classified within the Plecotini tribe (Bogdanowicz et al. 1998), which predominantly includes species characterized by long or square‐shaped ears. However, the phylogenetic analysis based on certain mitochondrial and nuclear genes could not support the monophyly of the Plecotini tribe (Roehrs et al. 2010).

Initial studies often depended on a limited number of mitochondrial genes for phylogenetic analysis, which sometimes led to unstable conclusions due to the paucity of data. Progress in sequencing technologies facilitates the acquisition of complete mitochondrial genomes. As a key resource for phylogenetic and molecular evolutionary research, they are now frequently applied in invertebrate and vertebrate researches (Li et al. 2021; Guan et al. 2025; Tan et al. 2025). To date, only a single mitochondrial genome sequence for each of *B. beijingensis* and *B. capsica* has been published within the genus *Barbastella*. Wang and Abduriyim (2025) have corroborated the close phylogenetic relationship between *Barbastella* and the genus *Plecotus* through an analysis of the mitochondrial genome of *B. capsica*. Guan et al. (2025) developed an updated phylogenetic framework for the order Chiroptera by integrating mitochondrial genomes with nuclear genes. This study molecularly confirmed that *Barbastella*, *Plecotus*, and *Corynorhinus* are all members of the tribe Plecotini within the subfamily Vespertilioninae. However, the study relied solely on the mitochondrial genomic data of *B. beijingensis* from the genus *Barbastella*. At present, other species within this genus lack comprehensive mitochondrial genome data, which presents considerable challenges for accurate phylogenetic inference within the genus and for defining its taxonomic boundaries with adjacent genera.

To enhance the genetic information database of the genus and to elucidate its phylogenetic position within the Vespertilionidae family, this study undertook the sequencing and assembly of the mitochondrial genomes of *B. darjelingensis* (HEB24051) from Hebei Province, China, and *B. beijingensis* (SX22052) from Shanxi Province, China. By integrating the previously published sequence of *B. capsica* (PP963575) and additional sequence of *B. beijingensis* (PV591999), we conducted a comparative analysis of the mitochondrial genome structures of these three Barbastelle species in China and performed phylogenetic reconstruction using genomic data from closely related taxa. This study not only augments the molecular data resources of the genus *Barbastella* but also provides crucial mitochondrial genomic evidence to further address enduring long‐standing taxonomic controversies within the Vespertilionidae family.

## 2 Materials and Methods

### 2.1 Sampling

One male *B. beijingensis* (specimen ID: SX22052) in Shanxi Provinces and one male *B. darjelingensis* (specimen ID: HEB24051) in Hebei province were captured. Postcapture, the animals were ethically euthanized, and their organs and tissues were rapidly frozen in liquid nitrogen for later genomic DNA extraction. Bat handling adhered to the Bat Workers' Manual (Mitchell‐Jones and McLeish 2004), and received approval from the Academic Ethics Committee of Henan Normal University (HNSD‐2025BS‐1009).

### 2.2 Genomic DNA Extraction and Sequencing

Genomic DNA was extracted using the FlaPure Animal Tissue DNA Extraction Kit (DE712‐50, Beijing Genesand Biotech Co. Ltd). Genomic DNA (200 ng) was utilized to construct DNA libraries with the VAHTS Universal Plus DNA Library Prep Kit (CAT#NDM617‐02, Vazyme) standard procedures. High‐throughput sequencing was performed on the DNBSEQ‐T7 platform. Raw sequencing data were filtered and deduplicated using Fastp v0.23.2 (Chen et al. 2018) and evaluated for quality using FastQC v0.11.9 (Bioinformatics 2015). Mitochondrial genome assembly was conducted using GetOrganelle software v1.7.6.1 (Jin et al. 2020), and genome annotation was performed with MitoZ v3.6 (Meng et al. 2019). Postquality control sequencing data were aligned to the reference mitochondrial genome sequence (PP591999) using BWA v0.7.12‐r1039 (Li and Durbin 2009). The mapping rate exceeded 98%, the average sequencing depth was over 500X, and the coverage reached 100% (Table 1). Raw data were uploaded to the Genome Sequence Archive, and the annotated mitochondrial genome sequence was submitted to the GenBase at the National Bioinformation Center of China (https://ngdc.cncb.ac.cn/) (Chen et al. 2021; Bao et al. 2024).

**TABLE 1:** The quality control information for sequences.

Specimen	Mapping rate (%)	Average sequencing depth (X)	Coverage (%)
SX22052	100	548	100
HEB24051	98	14,286	100

### 2.3 The Analysis of Mitochondrial Genome Sequence Characteristics

The Sequence Manipulation Suite JavaScript (http://www.detaibio.com/sms2/codon_plot.html) (Stothard 2000) was utilized to calculate the base composition ratios of the mitochondrial genome, including protein‐coding genes (PCGs), tRNA, rRNA, and the D‐loop region, as well as the AT and GC ratios and biases (Perna and Kocher 1995). Codon usage preference for each PCG was evaluated by calculating the relative synonymous codon usage (RSCU) using DnaSP v5 software (Librado and Rozas 2009). The nonsynonymous mutation frequency (*K*_*a*_) to synonymous mutation frequency (*K*_s_) nucleotide substitution rate (*K*_a_/*K*_s_) was calculated for each coding gene to assess selection pressure. The *K*_a_/*K*_s_ ratio assesses gene differentiation: a ratio > 1 indicates positive selection, a ratio < 1 indicates purifying selection, and a ratio = 1 suggests neutral evolution (Wang et al. 2009). The *K*_a_/*K*_s_ values for each gene were obtained through comparative analyses among *Barbastella* bats. The tRNA secondary structure was predicted using the online tool tRNAscan‐SE (https://lowelab.ucsc.edu/tRNAscan‐SE/) and visualized on the ViennaRNA website (http://rna.tbi.univie.ac.at/forna/). The structural characteristics of the D‐loop region were derived by referencing previously reported data on Chiroptera species (Sun et al. 2009; Rahman et al. 2019; Barrera et al. 2023; Martínez‐Cárdenas et al. 2024; Valencia et al. 2024).

### 2.4 Phylogenetic Analysis of the Family Vespertilionidae

The mitochondrial genomes from 23 species of Vespertilionidae were obtained from the National Center for Biotechnology Information (NCBI, https://www.ncbi.nlm.nih.gov/) to construct phylogenetic trees, with *Myotis davidii and M. macrodactylus* serving as the outgroup (Table 2). Thirteen PCGs from each species were concatenated and aligned using Geneious Pro v4.8.2 software (Biomatters Ltd., Auckland, New Zealand). Utilizing Mega v11.0 (Tamura et al. 2021), and the optimal nucleotide substitution model was identified as GTR + G + I *.* Maximum Likelihood (ML) phylogenetic analysis of the concatenated sequences was performed using RAxML‐HPC BlackBox v8.1.24 on the CIPRES Science Gateway platform (https://www.phylo.org/), employing 1000 bootstrap replications with values between 0 and 100. Bayesian Inference (BI) analysis was conducted using MrBayes v3.2.7 (Ronquist et al. 2012), which involved running four Markov chains with random starting trees for 1000 000 million generations, yielding posterior probability values ranging from 0 to 1. The resulting ML and BI phylogenetic trees were visualized using FigTree v1.4.4 (http://tree.bio.ed.ac.uk/software/figtree/).

**TABLE 2:** The accession numbers of mitochondrial genome of each species in this study.

Species	Accession number	Species	Accession number
*Barbastella beijingensis* (SX22052)	C_AA120226	*Nyctalus plancyi*	NC_041160
*Barbastella beijingensis*	PV591999	*Pipistrellus coromandra*	NC_029191
*Barbastella caspica*	PP963575	*Pipistrellus hanaki*	NC_029191
*Barbastella darjelingensis* (HEB24051)	C_AA120153	*Plecotus auritus*	NC_015484
*Bauerus dubiaquercus*	PQ064114	*Plecotus macrobullaris*	NC_027977
*Chalinolobus tuberculatus*	NC_002626	*Rhogeessa mira*	NC_083922
*Corynorhinus mexicanus*	PP472486	*Rhogeessa parvula*	NC_082319
*Corynorhinus rafinesquii*	NC_016872	*Tylonycteris fulvida*	MZ457524
*Eptesicus bottae*	NC_070014	*Vespertilio murinus*	NC_033347
*Eptesicus nilssonii*	NC_084105	*Vespertilio sinensis*	NC_024558
*Hypsugo alaschanicus*	NC_029939	*Myotis davidii*	NC_025568
*Lasiurus borealis*	NC_016873	*Myotis macrodactylus*	NC_022694
*Nyctalus noctula*	NC_027237		

## 3 Results

### 3.1 The Mitochondrial Genome Structures and Characteristics

The mitochondrial genomes of *B. beijingensis* (SX22052) and *B. darjelingensis* (HEB24051) have been assembled and annotated. SX22052 has a genome length of 16,667 bp, slightly longer than the16 590 bp listed in the NCBI database (PV591999), while *B. darjelingensis* has a total length of 16,434 bp, rendering both species shorter than *B. capsica* (PP963575, 16,933 bp). These length differences are mainly due to variations in the D‐loop region (Table S1). Since SX22052 closely matches the NCBI reference sequence (PV591999) in gene composition and arrangement, further analyses will focus on SX22052. Data on *B. capsica* is sourced from Wang and Abduriyim (2025).

**FIGURE 1:**
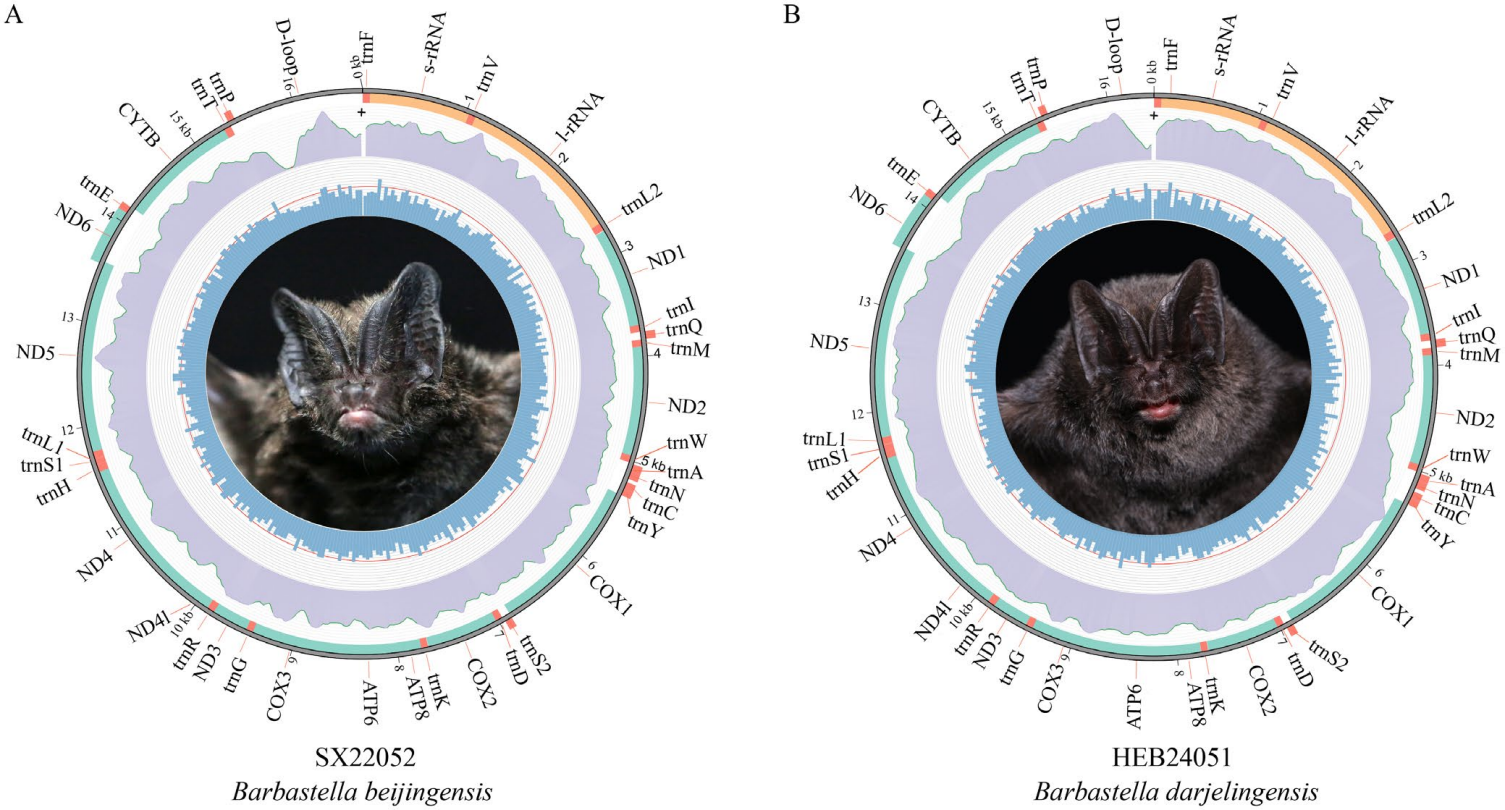
The mitochondrial genome maps of *Barbastella beijingensis* (A) and *B. darjelingensis* (B).

The mitochondrial genomes of *B. beijingensis* and *B. darjelingensis* each include 13 PCGs, 22 tRNA genes, 2 rRNA genes, and a D‐loop (Figure 1). Most genes are on the heavy strand, except for *ND6* and eight tRNA genes (*trnA*, *trnC*, *trnE*, *trnN*, *trnQ*, *trnP*, *trnS2*, and *trnY*), which are on the light strand. Genes may be contiguous, overlap, or be separated by intergenic regions, with overlaps ranging from 1 to 10 bp and the largest at 43 bp between *ATP8* and *ATP6*. Intergenic regions range from 1 to 7 bp, with the longest at 31–32 bp between *trnN* and *trnC* (Table S1). The mitochondrial genomes of the three species share similar nucleotide compositions (A > T > C > G) (Table S2) and exhibit strand asymmetry, with a positive AT bias and negative GC bias.

### 3.2 Comparative Analysis of Mitochondrial Protein‐Coding Genes

The combined length of the 13 PCGs is 11,452 bp for *B. beijingensis* , 11,445 bp for *B. darjelingensis*, and 11,408 bp for *B. capsica*. The shortest PCG is *ATP8* at 204 bp, while the longest is *ND5*, with *B. beijingensis* at 1833 bp and both *B. capsica* and *B. darjelingensis* at 1821 bp each (Table S1). The nucleotide composition of these PCGs follows the typical mitochondrial genome pattern, with the highest A ratio and lowest G ratio, and an AT ratio exceeding the GC ratio (Table S2). The first and third positions reflect the overall nucleotide composition trend, but the second position has the highest T ratio, indicating a negative AT bias.

Table S1 shows variations in start and stop codon usage among the species. The start codon is mainly AUG for 10 PCGs, while *ND2*, *ND3*, and *ND5* use AUA. These species use four stop codons: UAA, AGG, UAG, and AGA. UAA and UAG are the most common; AGA is exclusive to the *CYTB* gene, and AGG is unique to the *ND4* gene of *B. darjelingensis*. Additionally, certain genes in *B. beijingensis* and *B. capsica* exhibit incomplete stop codons in the form of “UA” or “U”.

**FIGURE 2:**
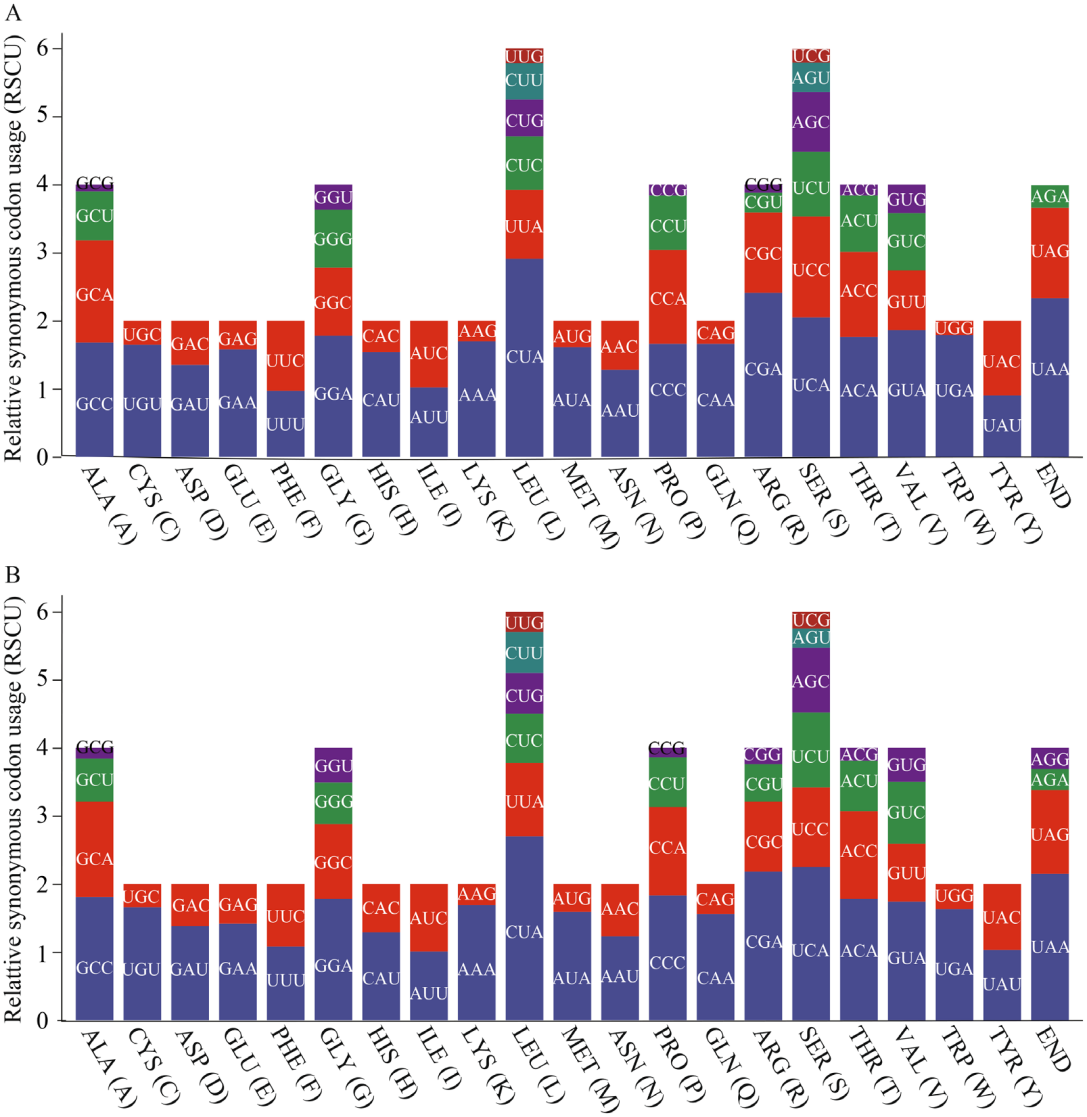
Relative synonymous codon usage (RSCU) for the PCGs of *Barbastella beijingensis* (A) and *B. darjelingensis* (B).

All 13 PCGs use multiple codons per amino acid, showing species‐specific codon usage variations but a consistent overall pattern (Figure 2). The leucine CUA codon is most frequent, appearing 291 times in *B. beijingensis* and 270 times in *B. darjelingensis* (Table S3). Common codons like AUA, AUU, AUC, and ACA occur over 140 times. In *B. capsica*, AAU, ACA, CCA, ACU, and CUA are the most frequent (Wang and Abduriyim 2025). The least used codons vary by species: CGG for arginine in *B. beijingensis* and *B. darjelingensis*, and GCG for alanine in *B. capsica*.

**FIGURE 3:**
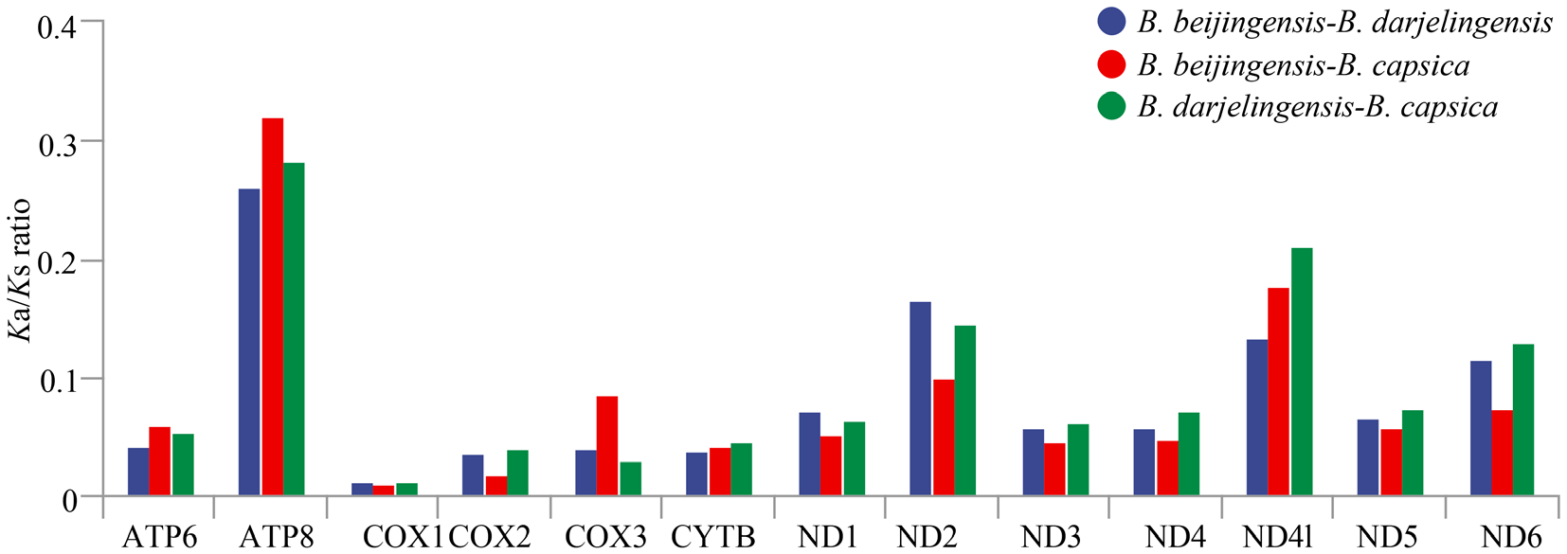
Analysis of the *K*_a_/*K*_s_ for the PCGs of *Barbastella beijingensis* and *B. darjelingensis*.

*K*_*a*_/*K*_*s*_ ratios for all PCGs in the three species are below 1 (Figure 3), indicating purifying selection. Gene conservation levels vary, with *ATP8* being less constrained (*K*_a_/*K*_s_ > 0.2), while genes linked to the cytochrome oxidase respiratory chain complex (*COX1*, *COX2*, and *COX3*) are under strong purifying selection due to low *K*_a_/*K*_s_ ratios.

### 3.3 Conserved Characteristics of Mitochondrial tRNA and rRNA

As shown in Table S1, the total tRNA length in *B. beijingensis* and *B. capsica* is 1508 bp, while tRNA in *B. darjelingensis* has a length of 1511 bp. The shortest rRNA gene is *trnS1* (59–60 bp), and the longest is *trnL2* (75 bp). All three species share a similar nucleotide composition (A > T > G > C) with a positive GC bias (Table S2).

Their tRNA secondary structures are mostly similar, with minor interspecies differences of 1–2 bp in certain arm regions (Table S4). Most tRNA genes have a classic cloverleaf structure, as shown in Figure S1 ( *B. beijingensis* ) and Figure S2 (*B. darjelingensis*), and Wang and Abduriyim (2025) for *B. capsica*. The *trnS1* gene lacks a D arm, setting it apart from other tRNAs. Base mismatches (A‐A, A‐C, A‐G, U‐U) are common across three species.

The 12S rRNA gene (*rrnS*) is located between *trnF* and *trnV*, and the 16S rRNA gene (*rrnL*) is between *trnV* and *trnL2*. The 12S rRNA lengths in *B. beijingensis* , *B. darjelingensis*, and *B. capsica* are 961 bp, 962 bp, and 960 bp, respectively, while the 16S rRNA lengths are 1566 bp, 1560 bp, and 1568 bp, showing high similarity in length and base composition, with notable positive AT bias (Table S2).

### 3.4 Structure and Tandem Repeats Variation of D‐Loop

**FIGURE 4:**
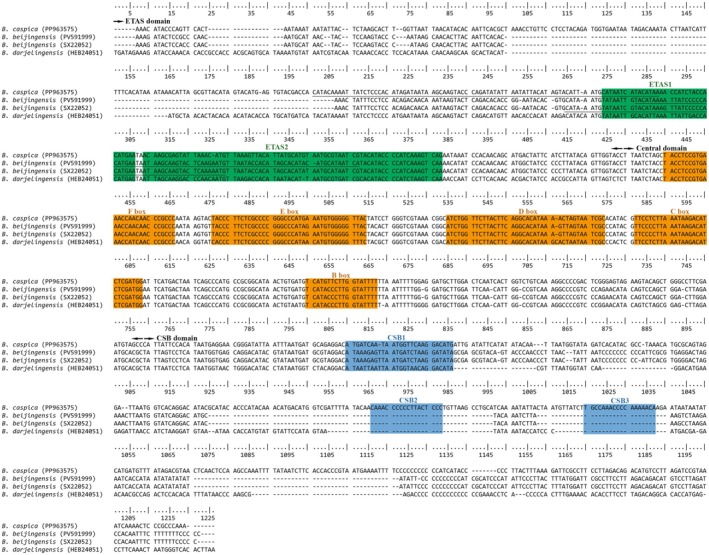
Sequence alignment for the 4 complete mitochondrial control regions of *Barbastella capsica* (PP963575), *B. beijingensis* (PV591999. SX22052), and *B. darjelingensis* (HEB24051). Conserved domains are color‐coded as follows: ETAS in blue, CD in yellow, and CSB in green. The underlined sections indicate tandem repeats.

In these Barbastelle bats, the D‐loop region, located between the *trnP* and *trnF* genes, measures 1232 bp, 1001 bp, and 1492 bp, respectively, contributing to mitochondrial genome size differences. The D‐loop regions are divided into the typical Extended Termination Associated Sequences (ETAS), Central Domain (CD), and Conserved Sequence Block (CSB) domains (Figure 4). *B. capsica* consistently has CSB1‐CSB3, whereas *B. beijingensis* and *B. darjelingensis* only have CSB1. The D‐loop region's base composition is similar to the overall mitochondrial genome (Table S2), with the highest A ratio, lowest G ratio, and a higher AT ratio than GC. The AT bias is positive, and the GC bias is negative. In the CD region of the three species, A, T, and C proportions are similar, with G being the least common, leading to a negative AT bias. *B. beijingensis* and *B. capsica* have long tandem repeats (80 bp) in the ETAS region, repeating 5.3 and 5.1 times, respectively. However, there is no tandem repeat in *B. darjelingensis*.

### 3.5 Phylogenetic Placement of Barbastelle Bats

**FIGURE 5:**
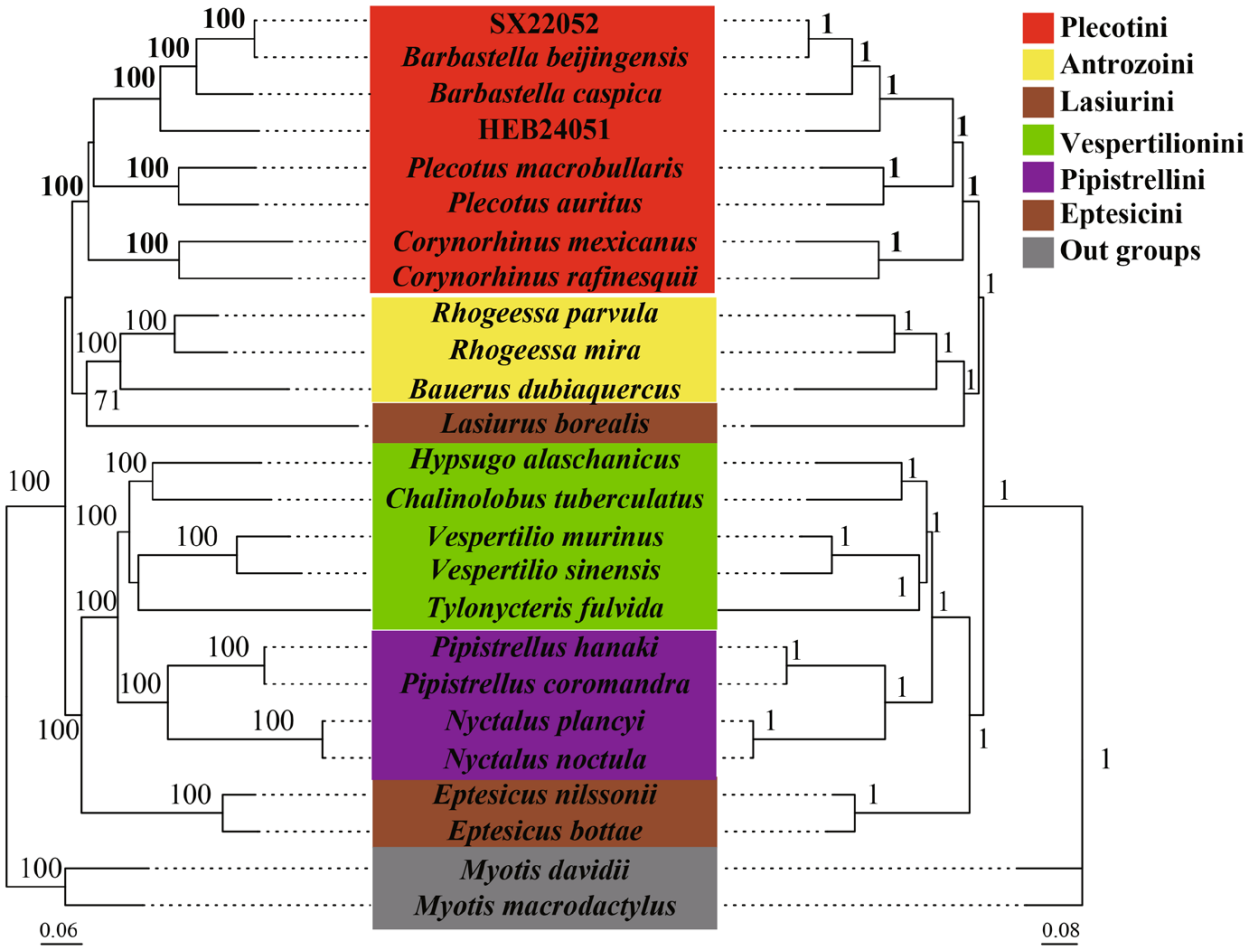
Phylogenetic analysis of *Barbastella* species and other Vespertilioninae species using 13 PCGs. The left side features a tree constructed through maximum‐likelihood (ML), while the right side shows a tree based on Bayesian inference (BI). *Myotis davidii* and *M* *yotis macrodactylus* are the outgroups, and nodes with bootstrap support values greater than 70 (ML) and posterior probabilities above 0.7 (BI) are indicated.

To elucidate the phylogenetic position of samples SX22052 and HEB24051 within the family Vespertilionidae, we acquired mitochondrial genome sequences from 23 species representing 14 genera within this family from the NCBI database. The ML and BI phylogenetic trees exhibited significant topological consistency (Figure 5). The genera *Barbastella*, *Plecotus*, and *Corynorhinus* initially clustered into a single clade, forming the Plecotini tribe, with *Barbastella* exhibiting the closest genetic relationship to *Plecotus*. Subsequently, the Plecotini tribe merged with the Antrozoini tribe (encompassing *Rhogeessa* and *Bauerus*) and the Lasiurini tribe (including *Lasiurus*) to form a larger clade. This clade is the sister group to another assemblage comprising Vespertilionini, Pipistrellini, and Eptesicini tribes, collectively constituting the Vespertilioninae subfamily. Most nodes within the phylogenetic trees demonstrated strong support, with 19 nodes exhibiting a bootstrap support value of 100 and a posterior probability of 1.00, indicating a highly reliable phylogenetic relationship.

## 4 Discussion

This study presents the complete mitochondrial genome sequences and their characteristics for two Barbastelle species, *B. beijingensis* and *B. darjelingensis*, with the sequence of *B. darjelingensis* being the first to be reported for the first time. Similar to the previously reported mitochondrial genome of *B. capsica*, the mitochondrial genomes of these two species exhibit a high degree of structural and functional similarity to other documented Chiroptera species (Meganathan et al. 2012; Chung et al. 2018; Vivas‐Toro et al. 2021; Barrera et al. 2023; Martínez‐Cárdenas et al. 2024; Valencia et al. 2024). These similarities encompass gene types, length, quantity, sequencing, base composition, PCG codon usage frequency, tRNA secondary structure and base mismatches, rRNA length, and D‐loop composition. This further underscores the high conservation of mitochondrial genomes within Chiroptera.

Notably, interspecies differences in the D‐loop region may be more pronounced. For example, within the CSB region, *B. beijingensis* and *B. darjelingensis* lack CSB2 and CSB3. The CSB region is critical for regulating both heavy‐strand and light‐strand transcription, highlighting its functional significance (Clayton 1991). However, in mammals, CSB2 and CSB3 are frequently absent across various species. CSB1 is consistently present and is considered essential, potentially playing a critical role in primer generation (Sbisà et al. 1997). In addition, the occurrence of tandem repeat sequences is likely attributable to pauses and slippage mispairing during heavy‐strand replication (Fumagalli et al. 1996), which may influence the regulation of mitochondrial genome replication and transcription (Delarbre et al. 2001). However, unlike *B. beijingensis* and *B. capsica*, which possess an 80‐bp tandem repeat in the ETAS region—a common feature among most bat species (Wilkinson et al. 1997), *B. darjelingensis* has lost these tandem repeats, similar to the situation observed in *Corynorhinus mexicanus* (Valencia et al. 2024), although the underlying mechanism remains unknown.

The analysis of selection pressure revealed that PCGs associated with the cytochrome oxidase respiratory chain complex (*COX1*, *COX2*, and *COX3*) are subject to strong purifying selection, whereas *ATP8* is less constrained. This pattern is common among the families Rhinolophidae, Phyllostomidae, Pteropodidae, and Vespertilionidae (Meganathan et al. 2012; Vivas‐Toro et al. 2021; Valencia et al. 2024). The COX genes function as the terminal enzyme in the mitochondrial electron transport chain, where they transfer electrons to oxygen, the ultimate electron acceptor (Čunátová et al. 2020). Their relatively rare nonsynonymous substitutions may play a crucial role in maintaining the essential function of mitochondrial energy metabolism. *ATP8* encodes a core subunit of the F0 component of ATPase. Within the mammalian mitochondrial genome, *ATP8* exhibits numerous highly variable sites, suggesting that its regulatory role may vary across species (da Fonseca et al. 2008), potentially resulting in weaker purifying selection, as observed in the Barbastelle bats.

The Vespertilionidae family, characterized by the highest species richness, presents a contentious internal taxonomic status. Currently, the family is classified into four subfamilies: Kerivoulinae, Murininae, Myotinae, and Vespertilioninae. The Vespertilioninae subfamily is further divided into nine tribes, including the Plecotini. Phylogenetic analyses employing maximum likelihood and Bayesian trees, constructed from tandem sequences of 13 PCGs, robustly support the classification of Barbastelle bats within this family. Specifically, three Barbastelle species are assigned to the Plecotini tribe of the Vespertilioninae subfamily, rather than to other tribes within this subfamily.

While this study incorporated data from three Barbastelle species, it encountered objective limitations due to data availability. Specifically, the mitochondrial genome information for many tribes remains undisclosed, complicating efforts to thoroughly and equitably elucidate phylogenetic relationships across all tribes. Methodologically, mitochondrial genomes offer substantial genetic variation information for analyzing the phylogeny of the order Chiroptera. However, it is important to acknowledge that mitochondrial genomes are maternally inherited and nonrecombinant, which can sometimes result in biased topological inferences when addressing complex evolutionary questions. Consequently, future research aiming to more accurately clarify elucidate the phylogenetic relationships and taxonomic status within Vespertilioninae should integrate mitochondrial genome data with nuclear gene data for joint analysis. This multievidence integration strategy can effectively mitigate the limitations associated with single genetic markers, thereby providing a closer approximation of the true evolutionary history of species.

## Author Contributions

**Sen Liu:** conceptualization (lead), data curation (lead), funding acquisition (lead), investigation (lead), methodology (lead), supervision (lead), validation (lead), writing – original draft (lead), writing – review and editing (equal). **Xinyu Yue:** formal analysis (lead), investigation (equal), writing – original draft (equal). **Hang Li:** formal analysis (equal), investigation (equal), validation (lead), visualization (equal). **Yangguang Sun:** investigation (equal), visualization (equal). **Xufan Wang:** investigation (equal), visualization (equal). **Dongge Guo:** funding acquisition (equal), investigation (lead). **Ying Wang:** conceptualization (equal), supervision (equal), writing – original draft (equal), writing – review and editing (lead).

## Conflicts of Interest

The authors declare no conflicts of interest.

## Supporting information


**Figure S1:** Secondary structures of the tRNAs of *Barbastella beijingensis* .

**Figure S2:** Secondary structures of the tRNAs of *Barbastella darjelingensis*.

**Table S1:** Annotation and gene order in the mitochondrial genome of Barbastelle bats distributed in China.

**Table S2:** Comparison of nucleotide composition of Barbastelle bats distributed in China.

**Table S3:** Frequency and RSCU values of codon in PCGs of *Barbastella beijingensis* and *B. darjelingensis*.

**Table S4:** Comparison of the length of the four stems that conform the secondary structure of the tRNAs of three Barbastelle species.

## Data Availability

All sequences associated with this research have been deposited into the Genome Sequence Archive (https://ngdc.cncb.ac.cn/gsa/, search GSA ID: CRA030092) and the GenBase (https://ngdc.cncb.ac.cn/genbase/?lang=en, search C_AA120226 for *Barbastella beijingensis* and C_AA120153 for *Barbastella darjelingensis*) in the National Bioinformation Center of China.
