# Supplementary material for: Comparative Analysis of Mitochondrial Genomes and Phylogeny of Barbastelle Bats Across China
Source: Ecol Evol. 2026 Jan 12;16(1):e72949. doi: 10.1002/ece3.72949 (PMC12793785; doi:10.1002/ece3.72949)
Supplement: Supplementary file 1 — Figure S1: Secondary structures of the tRNAs of Barbastella beijingensis . [file ECE3-16-e72949-s005.pdf]

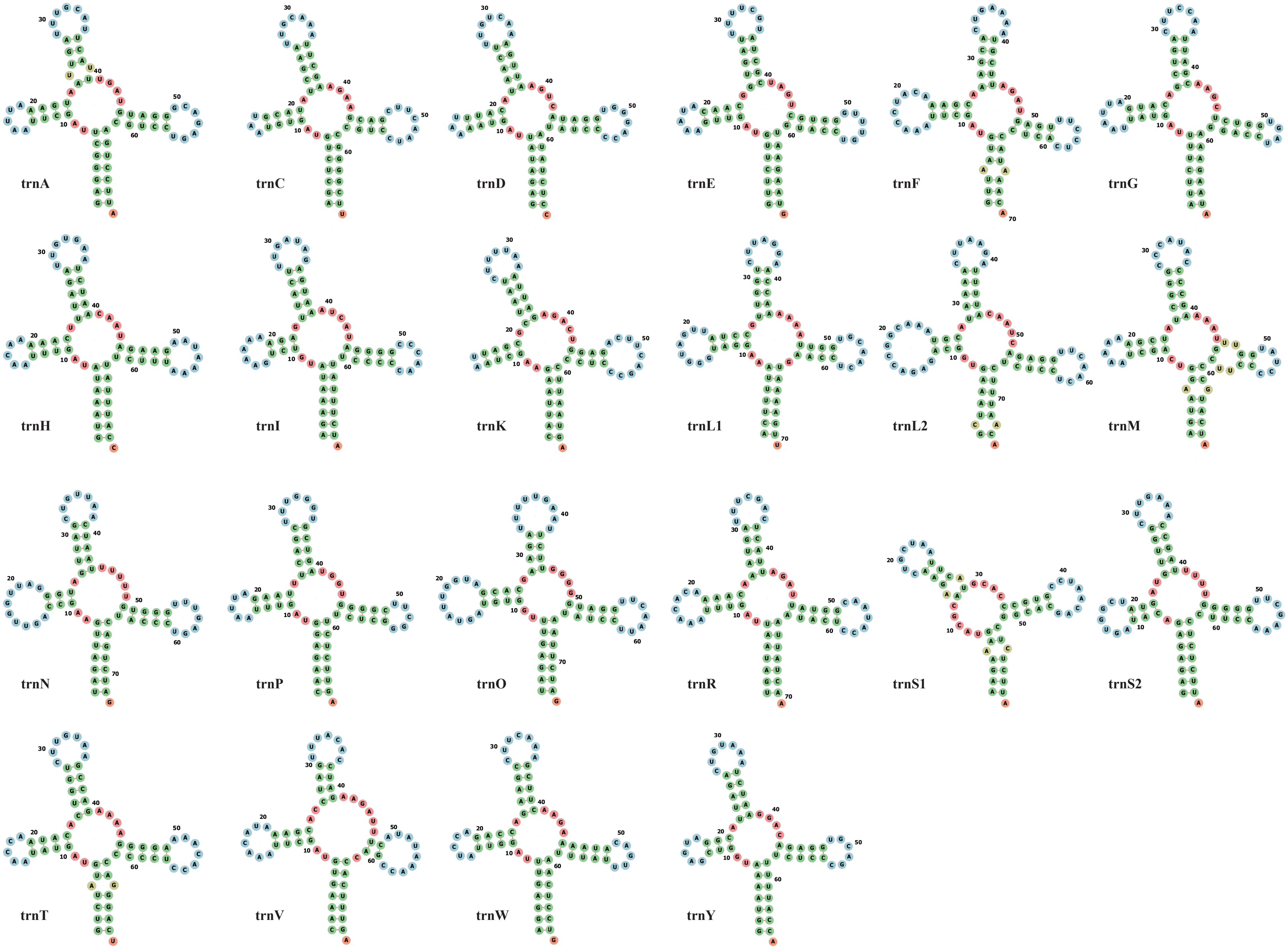
trnA
trnC
trnD
trnE
trnF
trnG
trnH
trnI
trnK
trnL1
trnL2
trnM
trnN
trnP
trnQ
trnR
trnS1
trnS2
trnT
trnV
trnW
trnY
