# Supplementary figures and images for: Comparative Analysis of Mitochondrial Genomes and Phylogeny of Barbastelle Bats Across China
Source: Ecol Evol. 2026 Jan 12;16(1):e72949. doi: 10.1002/ece3.72949 (PMC12793785; doi:10.1002/ece3.72949)

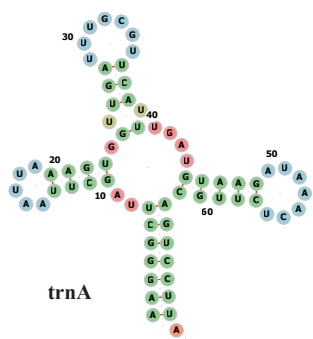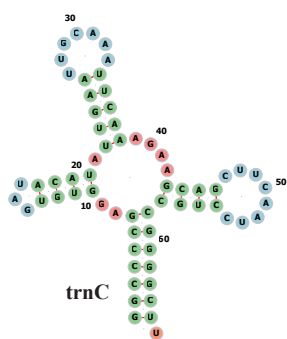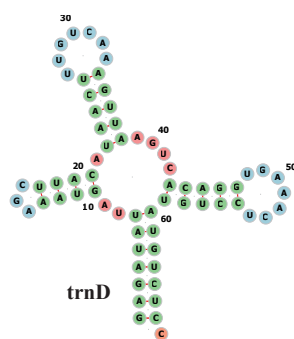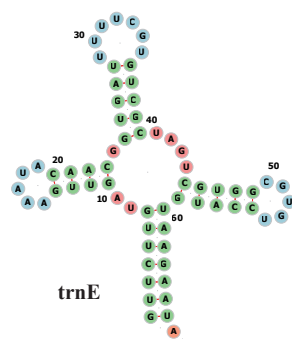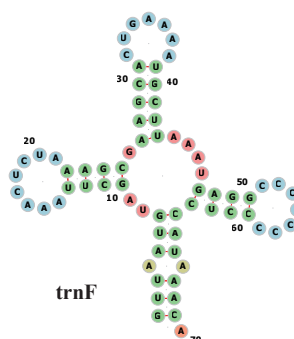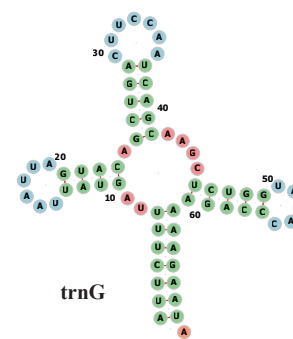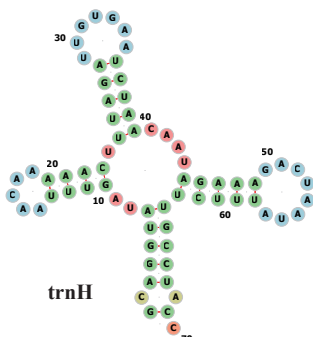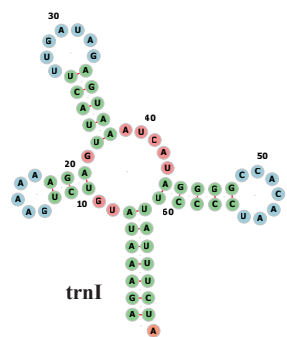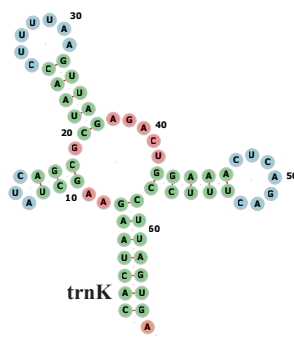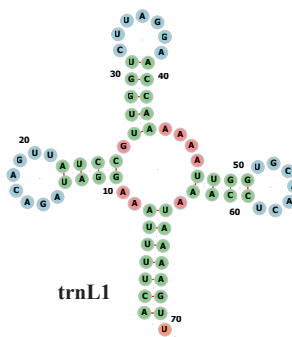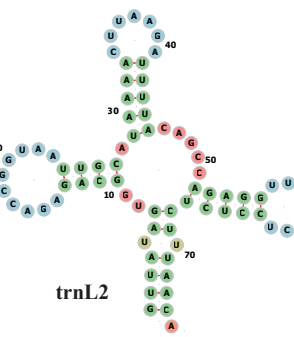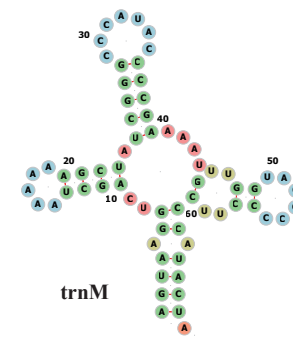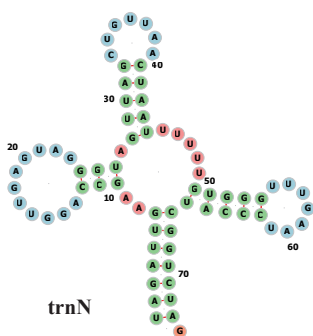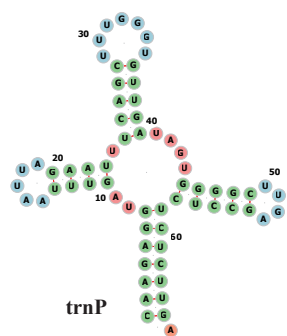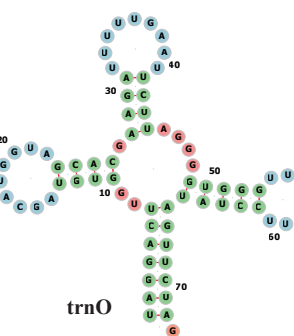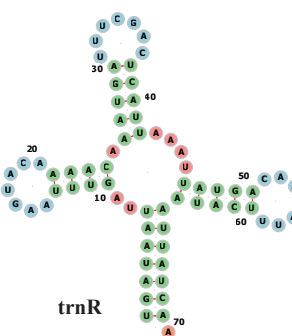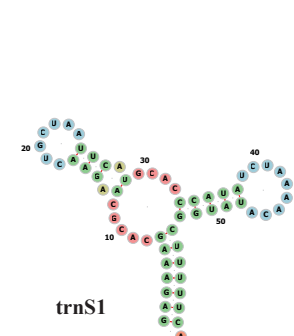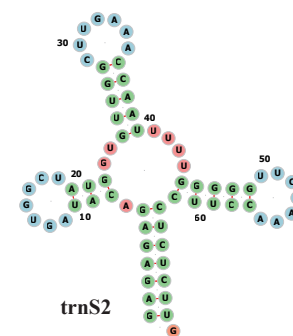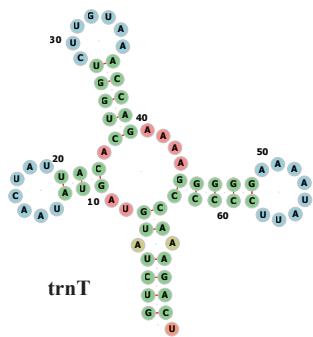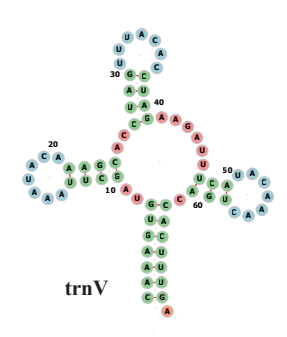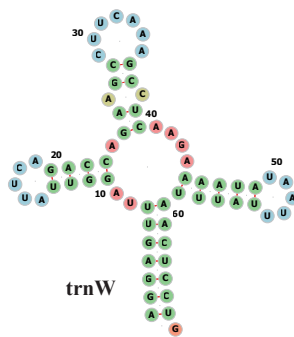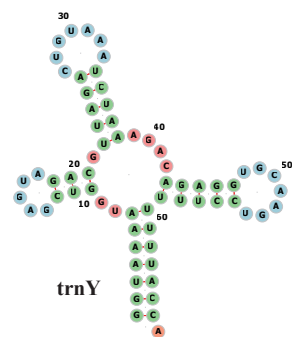

Supplement: Supplementary file 2 — Figure S2: Secondary structures of the tRNAs of Barbastella darjelingensis. [file ECE3-16-e72949-s003.pdf]
