# Supplementary material for: Comparative Analysis of Mitochondrial Genomes and Phylogeny of Barbastelle Bats Across China
Source: Ecol Evol. 2026 Jan 12;16(1):e72949. doi: 10.1002/ece3.72949 (PMC12793785; doi:10.1002/ece3.72949)
Supplement: Supplementary file 3 — Table S1: Annotation and gene order in the mitochondrial genome of Barbastelle bats distributed in China. [file ECE3-16-e72949-s004.docx]

**Table S1 Annotation and gene order in the mitochondrial genome of Barbastelle bats distributed in China**

| Gene name | Type | Direction | *Barbastella beijingensis* (SX22052) | | | *Barbastella darjelingensis* (HEB24051) | | | *Barbastella caspica* (PP963575) | | |
| --- | --- | --- | --- | --- | --- | --- | --- | --- | --- | --- | --- |
|  |  |  | Start-End (Length) | Codon(start/stop) | Continuity | Start-End (Length) | Codon(start/stop) | Continuity | Start-End (Length) | Codon(start/stop) | Continuity |
| *trnF* | tRNA | H | 1-70 (70) |  | 0 | 1-70 (70) |  | 0 | 1-72 (72) |  | 0 |
| *rrnS* (12S) | rRNA | H | 71-1031 (961) |  | 0 | 71-1032 (962) |  | 0 | 72-1031 (960) |  | -1 |
| *trnV* | tRNA | H | 1032-1100 (69) |  | 0 | 1033-1101 (69) |  | 0 | 1032-1100 (69) |  | 0 |
| *rrnL* (16S) | rRNA | H | 1100-2665 (1566) |  | -1 | 1101-2660 (1560) |  | -1 | 1101-2668 (1568) |  | 0 |
| *trnL2* | tRNA | H | 2667-2741 (75) |  | 1 | 2662-2736 (75) |  | 1 | 2669-2743 (75) |  | 0 |
| *ND1* | CDS | H | 2747-3703 (957) | AUG/UAA | 5 | 2742-3698 (957) | AUG/UAA | 5 | 2749-3705 (957) | AUG/UAA | 5 |
| *trnI* | tRNA | H | 3703-3770 (68) |  | -1 | 3698-3765 (68) |  | -1 | 3705-3772 (68) |  | -1 |
| *trnQ* | tRNA | L | 3768-3841 (74) |  | -3 | 3763-3835 (73) |  | -3 | 3770-3843 (74) |  | -3 |
| *trnM* | tRNA | H | 3842-3908 (67) |  | 0 | 3836-3903 (68) |  | 0 | 3844-3911 (68) |  | 0 |
| *ND2* | CDS | H | 3909-4952 (1044) | AUA/UAG | 0 | 3904-4947 (1044) | AUA/UAG | 0 | 3912-4953 (1042) | AUA/U-- | 0 |
| *trnW* | tRNA | H | 4951-5017 (67) |  | -2 | 4946-5012 (67) |  | -2 | 4954-5020 (67) |  | 0 |
| *trnA* | tRNA | L | 5024-5092 (69) |  | 6 | 5019-5087 (69) |  | 6 | 5028-5095 (68) |  | 7 |
| *trnN* | tRNA | L | 5093-5165 (73) |  | 0 | 5088-5161 (74) |  | 0 | 5096-5168 (73) |  | 0 |
| *trnC* | tRNA | L | 5198-5263 (66) |  | 32 | 5194-5259 (66) |  | 32 | 5200-5266 (67) |  | 31 |
| *trnY* | tRNA | L | 5264-5329 (66) |  | 0 | 5260-5326 (67) |  | 0 | 5267-5332 (66) |  | 0 |
| *COX1* | CDS | H | 5331-6875 (1545) | AUG/UAA | 1 | 5328-6872 (1545) | AUG/UAG | 1 | 5334-6878 (1545) | AUG/UAA | 1 |
| *trnS2* | tRNA | L | 6879-6947 (69) |  | 3 | 6876-6944 (69) |  | 3 | 6882-6950 (69) |  | 3 |
| *trnD* | tRNA | H | 6955-7021 (67) |  | 7 | 6952-7018 (67) |  | 7 | 6958-7024 (67) |  | 7 |
| *COX2* | CDS | H | 7022-7705 (684) | AUG/UAG | 0 | 7019-7702 (684) | AUG/UAA | 0 | 7025-7708 (684) | AUG/UAA | 0 |
| *trnK* | tRNA | H | 7709-7775 (67) |  | 3 | 7706-7771 (66) |  | 3 | 7711-7779 (69) |  | 2 |
| *ATP8* | CDS | H | 7777-7980 (204) | AUG/UAA | 1 | 7773-7976 (204) | AUG/UAA | 1 | 7780-7983 (204) | AUG/UAA | 0 |
| *ATP6* | CDS | H | 7938-8618 (681) | AUG/UAA | -43 | 7934-8614 (681) | AUG/UAA | -43 | 7941-8621 (681) | AUG/UAA | -43 |
| *COX3* | CDS | H | 8618-9421 (804) | AUG/UAG | -1 | 8614-9417 (804) | UTG/UAG | -1 | 8621-9404 (784) | AUG/U-- | -1 |
| *trnG* | tRNA | H | 9402-9468 (67) |  | -20 | 9398-9465 (68) |  | -20 | 9404-9472 (69) |  | -1 |
| *ND3* | CDS | H | 9469-9825 (357) | AUA/UAG | 0 | 9466-9822 (357) | AUA/UAG | 0 | 9472-9818 (347) | AUA/UA- | -1 |
| *trnR* | tRNA | H | 9816-9886 (71) |  | -10 | 9813-9883 (71) |  | -10 | 9819-9889 (71) |  | 0 |
| *ND4L* | CDS | H | 9888-10184 (297) | AUG/UAA | 1 | 9885-10181 (297) | AUG/UAA | 1 | 9891-10187 (297) | AUG/UAA | 1 |
| *ND4* | CDS | H | 10178-11555 (1378) | AUG/U-- | -7 | 10175-11557 (1383) | AUG/AGG | -7 | 10181-11558 (1378) | AUG/U-- | -7 |
| *trnH* | tRNA | H | 11556-11624 (69) |  | 0 | 11553-11622 (70) |  | -5 | 11559-11627 (69) |  | 0 |
| *trnS1* | tRNA | H | 11625-11683 (59) |  | 0 | 11623-11682 (60) |  | 0 | 11628-11686 (59) |  | 0 |
| *trnL1* | tRNA | H | 11685-11755 (71) |  | 1 | 11684-11754 (71) |  | 1 | 11688-11758 (71) |  | 1 |
| *ND5* | CDS | H | 11756-13588 (1833) | AUA/UAA | 0 | 11755-13575 (1821) | AUA/UAA | 0 | 11759-13579 (1821) | AUA/UAA | 0 |
| *ND6* | CDS | L | 13560-14087 (528) | AUG/UAA | -29 | 13559-14086 (528) | AUG/UAA | -17 | 13563-14090 (528) | AUG/UAA | -17 |
| *trnE* | tRNA | L | 14088-14155 (68) |  | 0 | 14087-14153 (67) |  | 0 | 14091-14158 (68) |  | 0 |
| *CYTB* | CDS | H | 14161-15300 (1140) | AUG/AGA | 5 | 14159-15298 (1140) | AUG/AGA | 5 | 14164-15303 (1140) | AUG/AGA | 5 |
| *trnT* | tRNA | H | 15301-15369 (69) |  | 0 | 15299-15368 (70) |  | 0 | 15304-15375 (72) |  | 0 |
| *trnP* | tRNA | L | 15369-15435 (67) |  | -1 | 15368-15433 (66) |  | -1 | 15373-15441 (69) |  | -3 |
| D-loop |  | H | 15436-16667 (1232) |  | 0 | 15434-16434 (1001) |  | 0 | 15442-16933 (1492) |  | 0 |
