# Supplementary material for: Comparative Analysis of Mitochondrial Genomes and Phylogeny of Barbastelle Bats Across China
Source: Ecol Evol. 2026 Jan 12;16(1):e72949. doi: 10.1002/ece3.72949 (PMC12793785; doi:10.1002/ece3.72949)
Supplement: Supplementary file 4 — Table S2: Comparison of nucleotide composition of Barbastelle bats distributed in China. [file ECE3-16-e72949-s006.docx]

**Table S2 Comparison of nucleotide composition of Barbastelle bats distributed in China**

|  |  | G (%) | C (%) | A (%) | T (%) | G+C (%) | A+T (%) | GC-Skew | AT-Skew |
| --- | --- | --- | --- | --- | --- | --- | --- | --- | --- |
| Mitogenome | *B. beijingensis* (SX22052) | 13.9 | 26.9 | 33.4 | 25.8 | 40.8 | 59.2 | -0.319 | 0.128 |
|  | *B. darjelingensis* (HEB24051) | 14.3 | 26.7 | 33.0 | 26.0 | 41.0 | 59.0 | -0.302 | 0.119 |
|  | *B. capsica* (PP963575) | 13.5 | 26.4 | 33.9 | 26.2 | 39.9 | 60.1 | -0.323 | 0.128 |
| PCGs | *B. beijingensis* (SX22052) | 13.7 | 27.3 | 31.0 | 28.0 | 41.0 | 59.0 | -0.332 | 0.051 |
|  | *B. darjelingensis* (HEB24051) | 14.2 | 27.0 | 30.5 | 28.3 | 41.2 | 58.8 | -0.311 | 0.037 |
|  | *B. capsica* (PP963575) | 13.4 | 27.2 | 31.2 | 28.2 | 40.6 | 59.4 | -0.340 | 0.051 |
| PCGs--1st | *B. beijingensis* (SX22052) | 21.0 | 24.5 | 32.2 | 22.3 | 45.5 | 54.5 | -0.077 | 0.182 |
|  | *B. darjelingensis* (HEB24051) | 21.5 | 24.2 | 32.1 | 22.2 | 45.7 | 54.3 | -0.059 | 0.182 |
|  | *B. capsica* (PP963575) | 21.0 | 23.9 | 32.6 | 22.5 | 44.9 | 55.1 | -0.065 | 0.183 |
| PCGs--2st | *B. beijingensis* (SX22052) | 12.5 | 26.4 | 19.6 | 41.5 | 38.9 | 61.1 | -0.357 | -0.358 |
|  | *B. darjelingensis* (HEB24051) | 12.5 | 26.4 | 19.7 | 41.4 | 38.9 | 61.1 | -0.357 | -0.355 |
|  | *B. capsica* (PP963575) | 12.4 | 26.5 | 19.5 | 41.6 | 38.9 | 61.1 | -0.362 | -0.362 |
| PCGs--3st | *B. beijingensis* (SX22052) | 7.8 | 30.9 | 41.0 | 20.3 | 38.7 | 61.3 | -0.597 | 0.338 |
|  | *B. darjelingensis* (HEB24051) | 8.8 | 30.3 | 39.9 | 21.0 | 39.1 | 60.9 | -0.550 | 0.310 |
|  | *B. capsica* (PP963575) | 6.9 | 31.1 | 41.6 | 20.4 | 38.0 | 62.0 | -0.637 | 0.342 |
| tRNA | *B. beijingensis* (SX22052) | 20.3 | 18.0 | 31.6 | 30.1 | 38.3 | 61.7 | 0.060 | 0.024 |
|  | *B. darjelingensis* (HEB24051) | 19.9 | 18.2 | 31.9 | 30.0 | 38.1 | 61.9 | 0.045 | 0.031 |
|  | *B. capsica* (PP963575) | 19.4 | 17.9 | 32.2 | 30.5 | 37.3 | 62.7 | 0.040 | 0.027 |
| rRNA | *B. beijingensis* (SX22052) | 17.7 | 23.2 | 37.4 | 21.7 | 40.9 | 59.1 | -0.134 | 0.266 |
|  | *B. darjelingensis* (HEB24051) | 18.4 | 22.6 | 36.6 | 22.4 | 41.0 | 59.0 | -0.102 | 0.241 |
|  | *B. capsica* (PP963575) | 18.0 | 23.1 | 37.1 | 21.8 | 41.1 | 58.9 | -0.124 | 0.260 |
| 12S rRNA | *B. beijingensis* (SX22052) | 18.9 | 23.5 | 36.4 | 21.2 | 42.4 | 57.6 | -0.108 | 0.264 |
|  | *B. darjelingensis* (HEB24051) | 19.3 | 23.3 | 36.0 | 21.4 | 42.6 | 57.4 | -0.094 | 0.254 |
|  | *B. capsica* (PP963575) | 19.1 | 23.5 | 36.1 | 21.3 | 42.6 | 57.4 | -0.103 | 0.258 |
| 16S rRNA | *B. beijingensis* (SX22052) | 17.1 | 22.9 | 38.0 | 22.0 | 40.0 | 60.0 | -0.145 | 0.267 |
|  | *B. darjelingensis* (HEB24051) | 17.8 | 22.1 | 37.1 | 23.0 | 39.9 | 60.1 | -0.108 | 0.235 |
|  | *B. capsica* (PP963575) | 17.3 | 22.8 | 37.7 | 22.2 | 40.1 | 59.9 | -0.137 | 0.259 |
| D-loop | *B. beijingensis* (SX22052) | 13.7 | 25.8 | 35.3 | 25.2 | 39.5 | 60.5 | -0.306 | 0.167 |
|  | *B. darjelingensis* (HEB24051) | 17.4 | 25.7 | 28.0 | 28.9 | 43.1 | 56.9 | -0.193 | -0.016 |
|  | *B. capsica* (PP963575) | 13.0 | 22.3 | 36.7 | 28.0 | 35.3 | 64.7 | -0.263 | 0.134 |
| D-loop-ETAS Domain | *B. beijingensis* (SX22052) | 9.7 | 24.7 | 42.6 | 23.0 | 34.4 | 65.6 | -0.436 | 0.299 |
|  | *B. darjelingensis* (HEB24051) | 10.7 | 27.0 | 40.8 | 21.5 | 37.7 | 62.3 | -0.432 | 0.310 |
|  | *B. capsica* (PP963575) | 9.2 | 19.1 | 42.7 | 29.0 | 28.3 | 71.7 | -0.350 | 0.191 |
| D-loop -Contral Domain | *B. beijingensis* (SX22052) | 20.6 | 27.5 | 25.6 | 26.3 | 48.1 | 51.9 | -0.143 | -0.013 |
|  | *B. darjelingensis* (HEB24051) | 20.3 | 28.6 | 25.2 | 25.9 | 48.9 | 51.1 | -0.170 | -0.014 |
|  | *B. capsica* (PP963575) | 21.7 | 25.6 | 25.6 | 27.1 | 47.3 | 52.7 | -0.082 | -0.028 |
| D-loop -CSB Domain | *B. beijingensis* (SX22052) | 14.3 | 26.4 | 31.0 | 28.3 | 40.7 | 59.3 | -0.297 | 0.046 |
|  | *B. darjelingensis* (HEB24051) | 14.3 | 27.2 | 35.8 | 22.7 | 41.5 | 58.5 | -0.311 | 0.224 |
|  | *B. capsica* (PP963575) | 12.8 | 24.8 | 35.6 | 26.8 | 37.6 | 62.4 | -0.319 | 0.141 |
