# Supplementary material for: Comparative Analysis of Mitochondrial Genomes and Phylogeny of Barbastelle Bats Across China
Source: Ecol Evol. 2026 Jan 12;16(1):e72949. doi: 10.1002/ece3.72949 (PMC12793785; doi:10.1002/ece3.72949)
Supplement: Supplementary file 5 — Table S3: Frequency and RSCU values of codon in PCGs of Barbastella beijingensis and B. darjelingensis. [file ECE3-16-e72949-s002.docx]

**Table S3 Frequency and RSCU values of codon in PCGs of *Barbastella beijingensis* and *B. darjelingensis***

| AA | Codon | *B. beijingensis* (SX22052) | | *B. darjelingensis* (HEB24051) | | AA | Codon | *B. beijingensis* (SX22052) | | *B. darjelingensis* (HEB24051) | |
| --- | --- | --- | --- | --- | --- | --- | --- | --- | --- | --- | --- |
|  |  | Count | RSCU | Count | RSCU |  |  | Count | RSCU | Count | RSCU |
| Ala(A) | GCG | 6 | 0.10 | 10 | 0.16 | Pro(P) | CCG | 8 | 0.16 | 7 | 0.14 |
|  | GCA | 91 | 1.50 | 87 | 1.40 |  | CCA | 71 | 1.38 | 66 | 1.30 |
|  | GCU | 44 | 0.72 | 39 | 0.63 |  | CCU | 41 | 0.80 | 37 | 0.73 |
|  | GCC | 102 | 1.68 | 112 | 1.81 |  | CCC | 85 | 1.66 | 93 | 1.83 |
| Cys(C) | UGU | 4 | 0.35 | 5 | 0.34 | Gln(Q) | CAG | 15 | 0.34 | 20 | 0.44 |
|  | UGC | 19 | 1.65 | 24 | 1.66 |  | CAA | 74 | 1.66 | 70 | 1.56 |
| Asp(D) | GAU | 21 | 0.65 | 21 | 0.62 | Arg(R) | CGG | 2 | 0.12 | 4 | 0.24 |
|  | GAC | 44 | 1.35 | 47 | 1.38 |  | CGA | 41 | 2.41 | 36 | 2.18 |
| Glu(E) | GAG | 20 | 0.42 | 28 | 0.58 |  | CGU | 5 | 0.29 | 9 | 0.55 |
|  | GAA | 75 | 1.58 | 69 | 1.42 |  | CGC | 20 | 1.18 | 17 | 1.03 |
| Phe(F) | UUU | 109 | 0.97 | 117 | 1.08 | Ser(S) | AGU | 21 | 0.43 | 13 | 0.28 |
|  | UUC | 115 | 1.03 | 99 | 0.92 |  | AGC | 43 | 0.88 | 45 | 0.95 |
| Gly(G) | GGG | 46 | 0.85 | 33 | 0.61 |  | UCG | 10 | 0.21 | 12 | 0.25 |
|  | GGA | 96 | 1.78 | 97 | 1.78 |  | UCA | 100 | 2.05 | 106 | 2.25 |
|  | GGU | 20 | 0.37 | 28 | 0.51 |  | UCU | 46 | 0.95 | 52 | 1.10 |
|  | GGC | 54 | 1.00 | 60 | 1.10 |  | UCC | 72 | 1.48 | 55 | 1.17 |
| His(H) | CAU | 22 | 0.46 | 37 | 0.71 | Thr(T) | ACG | 13 | 0.16 | 16 | 0.19 |
|  | CAC | 74 | 1.54 | 67 | 1.29 |  | ACA | 146 | 1.76 | 147 | 1.78 |
| Ile(I) | AUU | 161 | 1.02 | 160 | 1.01 |  | ACU | 69 | 0.83 | 61 | 0.74 |
|  | AUC | 156 | 0.98 | 157 | 0.99 |  | ACC | 104 | 1.25 | 107 | 1.29 |
| Lys(K) | AAG | 15 | 0.30 | 15 | 0.31 | Val(V) | GUG | 19 | 0.42 | 24 | 0.50 |
|  | AAA | 85 | 1.70 | 82 | 1.69 |  | GUA | 84 | 1.86 | 82 | 1.74 |
| Leu(L) | UUG | 22 | 0.22 | 29 | 0.30 |  | GUU | 40 | 0.88 | 40 | 0.85 |
|  | UUA | 101 | 1.01 | 106 | 1.08 |  | GUC | 38 | 0.84 | 43 | 0.91 |
|  | CUG | 54 | 0.54 | 58 | 0.60 | Trp(W) | UGG | 11 | 0.21 | 19 | 0.37 |
|  | CUA | 291 | 2.91 | 270 | 2.70 |  | UGA | 94 | 1.79 | 85 | 1.63 |
|  | CUU | 53 | 0.53 | 60 | 0.60 | Tyr(Y) | UAU | 61 | 0.90 | 66 | 1.03 |
|  | CUC | 79 | 0.79 | 71 | 0.72 |  | UAC | 75 | 1.10 | 62 | 0.97 |
| Met(M) | AUG | 51 | 0.39 | 54 | 0.41 | End | AGG | 0 | 0.00 | 1 | 0.31 |
|  | AUA | 211 | 1.61 | 210 | 1.59 |  | AGA | 1 | 0.33 | 1 | 0.31 |
| Asn(N) | AAU | 56 | 0.72 | 60 | 0.77 |  | UAG | 4 | 1.33 | 4 | 1.23 |
|  | AAC | 100 | 1.28 | 96 | 1.23 |  | UAA | 7 | 2.33 | 7 | 2.15 |
