# Supplementary material for: Comparative Analysis of Mitochondrial Genomes and Phylogeny of Barbastelle Bats Across China
Source: Ecol Evol. 2026 Jan 12;16(1):e72949. doi: 10.1002/ece3.72949 (PMC12793785; doi:10.1002/ece3.72949)
Supplement: Supplementary file 6 — Table S4: Comparison of the length of the four stems that conform the secondary structure of the tRNAs of three Barbastelle species. [file ECE3-16-e72949-s001.docx]

**Table S4 Comparison of the length of the four stems that conform the secondary structure of the tRNAs of three Barbastelle species**

|  | Aceptor stem length (bp) | | | Anticodon arm length (bp) | | | D arm length (bp) | | | TψC arm length (bp) | | |
| --- | --- | --- | --- | --- | --- | --- | --- | --- | --- | --- | --- | --- |
|  | *B. beijingensis*  SX22052 | *B. darjelingensis*  HEB24051 | *B. capsica*  PP963575 | *B. beijingensis*  SX22052 | *B. darjelingensis*  HEB24051 | *B. capsica*  PP963575 | *B. beijingensis*  SX22052 | *B. darjelingensis*  HEB24051 | *B. capsica*  PP963575 | *B. beijingensis*  SX22052 | *B. darjelingensis*  HEB24051 | *B. capsica*  PP963575 |
| trnF | 7 | 7 | 7 | 5 | 5 | 5 | 4 | 4 | 4 | 4 | 4 | 4 |
| trnV | 7 | 7 | 7 | 4 | 4 | 4 | 4 | 4 | 4 | 2 | 3 | 3 |
| trnL2 | 7 | 7 | 7 | 5 | 5 | 5 | 3 | 4 | 3 | 5 | 5 | 5 |
| trnI | 7 | 7 | 7 | 5 | 5 | 5 | 3 | 3 | 3 | 5 | 5 | 5 |
| trnQ | 7 | 7 | 7 | 4 | 4 | 4 | 4 | 4 | 4 | 5 | 5 | 5 |
| trnM | 7 | 7 | 7 | 5 | 5 | 5 | 4 | 4 | 4 | 5 | 5 | 5 |
| trnW | 7 | 7 | 7 | 5 | 5 | 5 | 4 | 4 | 4 | 5 | 5 | 5 |
| trnA | 7 | 7 | 7 | 5 | 5 | 5 | 4 | 4 | 4 | 5 | 5 | 5 |
| trnN | 7 | 7 | 7 | 5 | 5 | 5 | 3 | 3 | 3 | 5 | 5 | 5 |
| trnC | 7 | 7 | 7 | 5 | 5 | 5 | 4 | 4 | 4 | 4 | 4 | 4 |
| trnY | 7 | 7 | 7 | 5 | 5 | 5 | 3 | 3 | 3 | 5 | 5 | 5 |
| trnS2 | 7 | 7 | 7 | 5 | 5 | 5 | 3 | 3 | 3 | 5 | 5 | 5 |
| trnD | 7 | 7 | 7 | 5 | 5 | 4 | 4 | 4 | 4 | 5 | 5 | 5 |
| trnK | 7 | 7 | 7 | 5 | 5 | 5 | 4 | 3 | 4 | 5 | 5 | 5 |
| trnG | 7 | 7 | 7 | 5 | 5 | 5 | 4 | 4 | 4 | 5 | 5 | 5 |
| trnR | 7 | 7 | 7 | 5 | 5 | 5 | 4 | 4 | 4 | 5 | 5 | 5 |
| trnH | 7 | 7 | 7 | 5 | 5 | 5 | 4 | 4 | 4 | 5 | 5 | 5 |
| trnS1 | 7 | 7 | 5 | 5 | 5 | 5 | 0 | 0 | 0 | 5 | 5 | 5 |
| trnL1 | 7 | 7 | 7 | 5 | 5 | 5 | 4 | 4 | 4 | 4 | 4 | 4 |
| trnE | 7 | 7 | 7 | 5 | 5 | 5 | 4 | 4 | 4 | 5 | 5 | 5 |
| trnT | 7 | 7 | 7 | 5 | 5 | 5 | 4 | 3 | 3 | 5 | 5 | 5 |
| trnP | 7 | 7 | 7 | 5 | 5 | 5 | 4 | 4 | 4 | 5 | 5 | 5 |
